# Cardiac Magnetic Resonance-Supported Diagnosis of Acute Myocarditis Mimicking Acute Coronary Syndrome in a Patient With Sepsis Due to a Massive Perianal Abscess

**DOI:** 10.14740/jmc5410

**Published:** 2026-10-02

**Authors:** Blerim Arapi, Alert Xhaja, Rudin Domi, Ilir Sharka, Argjir Taka, Gentian Huti, Asead Abdyli, Alfred Nona, Krenar Lilaj, Alma Cani, Filadelfo Coniglione, Mustafa Bajraktari, Majlinda Naco, Erjona Sinani, Vedat Eljezi

**Affiliations:** a Department of Anesthesia and Intensive Care, Hygeia International Hospital, Tirana, Albania; b Department of Infection Diseases, Service of Dermatology, University of Medicine, Tirana, Albania; c Department of Surgery, Service of Anesthesia and Intensive Care, University of Medicine, Tirana, Albania; d Department of Anesthesia and Intensive Care, American Hospital 3, Tirana, Albania; e Department of Cardiology, Service of Cardiology, University of Medicine, Tirana, Albania; f Department of Cardiology, American Hospital 2, Tirana, Albania; g Department of Clinical Science and Translational Medicine, Tor Vergata University of Rome, Rome, Italy; h Department of Anesthesia and Intensive Care, American Hospital 2, Tirana, Albania; i Department of General Surgery, Hygeia International Hospital, Tirana, Albania; j Department of Perioperative Medicine, CHU Gabriel-Montpied, Clermont-Ferrand, France

**Keywords:** Acute myocarditis, Perianal abscess, Sepsis, Emergency surgery, Myocardial injury, Mitral regurgitation, Myocardial infarction with non-obstructive coronary arteries

## Abstract

Acute myocarditis is a rare but potentially life-threatening inflammatory disease that may mimic acute myocardial infarction, particularly in septic patients where myocardial injury may have other causes. We report the case of a 46-year-old man with sepsis secondary to a massive perianal abscess who underwent emergency surgical drainage under general anesthesia. Postoperatively, persistent respiratory deterioration and elevated cardiac biomarkers prompted further cardiac evaluation. Echocardiography showed newly reduced left ventricular (LV) systolic function, with an ejection fraction (EF) of 35%, and moderate-to-severe mitral regurgitation (MR). Although acute myocardial infarction was initially suspected, coronary angiography showed non-obstructive coronary arteries (MINOCA), with only a 40% right coronary artery stenosis. Cardiac magnetic resonance (CMR) demonstrated elevated T2 values and late gadolinium enhancement (LGE) of the interventricular septum, with additional enhancement of the LV lateral wall, consistent with myocardial edema and inflammation and supportive of acute myocarditis. The patient received source control, broad-spectrum antibiotics, intravenous diuretics, norepinephrine and dobutamine, oxygen therapy, and heart failure-directed therapy. EF improved to 45% at 10 days, with complete recovery of cardiac function and resolution of MR at the 3-month follow-up. This case highlights the diagnostic challenge of acute myocarditis in septic surgical patients and suggests that early multidisciplinary assessment, coronary evaluation, and CMR may help clarify the diagnosis and guide management.

## Introduction

Acute myocarditis is an inflammatory disease of the myocardium caused by infectious, immune-mediated, or toxic insults, with a highly variable clinical presentation ranging from asymptomatic myocardial injury to fulminant heart failure, malignant arrhythmias, cardiogenic shock, and sudden cardiac death. Although viral infections remain the most common cause in developed countries, bacterial infections and systemic inflammatory conditions may also trigger myocardial inflammation through direct or immune-mediated mechanisms [1, 2]. The diagnosis of acute myocarditis remains challenging because its clinical presentation frequently overlaps with acute coronary syndrome (ACS). Chest discomfort, electrocardiographic abnormalities, elevated cardiac biomarkers, and new regional or global ventricular dysfunction may closely resemble acute myocardial infarction. Coronary angiography may demonstrate non-obstructive coronary arteries, requiring integration of clinical findings, cardiac imaging, and, in selected cases, endomyocardial biopsy (EMB) to establish the diagnosis [3]. This challenge is particularly important in patients with severe sepsis, in whom myocardial injury and ventricular dysfunction are often attributed to sepsis-induced cardiomyopathy (SICM) or type 2 myocardial infarction related to oxygen supply–demand imbalance. Thus, myocarditis may remain unrecognized, particularly in the peri-operative period, when respiratory deterioration, hemodynamic instability, pulmonary edema, and biomarker elevation may have several competing explanations [4, 5]. A stepwise evaluation incorporating the clinical course, electrocardiography, cardiac biomarkers, echocardiography, coronary assessment, and cardiac magnetic resonance (CMR) can help distinguish myocarditis from ischemic injury and other forms of sepsis-associated myocardial dysfunction. Emergency surgery adds further complexity when myocardial dysfunction is not yet recognized. In this setting, anesthesia and peri-operative management require careful attention to cardiovascular stability, myocardial oxygen demand, preload and afterload, fluid administration, and the potential for acute deterioration during induction or emergence. These considerations must be balanced against the need for timely surgical source control in severe perianal infection, which is essential to prevent progression of local infection and overwhelming sepsis [6]. Close postoperative cardiopulmonary monitoring and early multidisciplinary re-assessment become particularly important when unexpected respiratory or cardiovascular abnormalities develop.

We report the case of a 46-year-old man with severe sepsis secondary to a massive perianal abscess who underwent emergency drainage under general anesthesia and subsequently developed acute respiratory deterioration, marked cardiac biomarker elevation, new left ventricular (LV) systolic dysfunction, and moderate-to-severe mitral regurgitation (MR). Acute myocardial infarction was initially suspected because of the cardiac biomarker and electrocardiographic abnormalities; however, coronary angiography demonstrated only non-obstructive coronary disease, while CMR subsequently provided evidence supporting acute myocarditis. This case highlights the diagnostic challenge of distinguishing myocarditis from sepsis-induced myocardial dysfunction and ACS in the immediate peri-operative period, and emphasizes the importance of stepwise cardiac evaluation, appropriate anesthetic and hemodynamic management, early multidisciplinary assessment, coronary exclusion of obstructive disease, and CMR-based tissue characterization in guiding diagnosis and treatment.

## Case Report

### Investigations

A 46-year-old man presented to the emergency department after a 10-day history of fever. On admission, he had a temperature of 40 °C, was mildly tachypneic and diaphoretic, and reported only a mild non-productive cough, fatigue, and pain associated with a large perianal abscess (Fig. 1).

**Figure 1:**
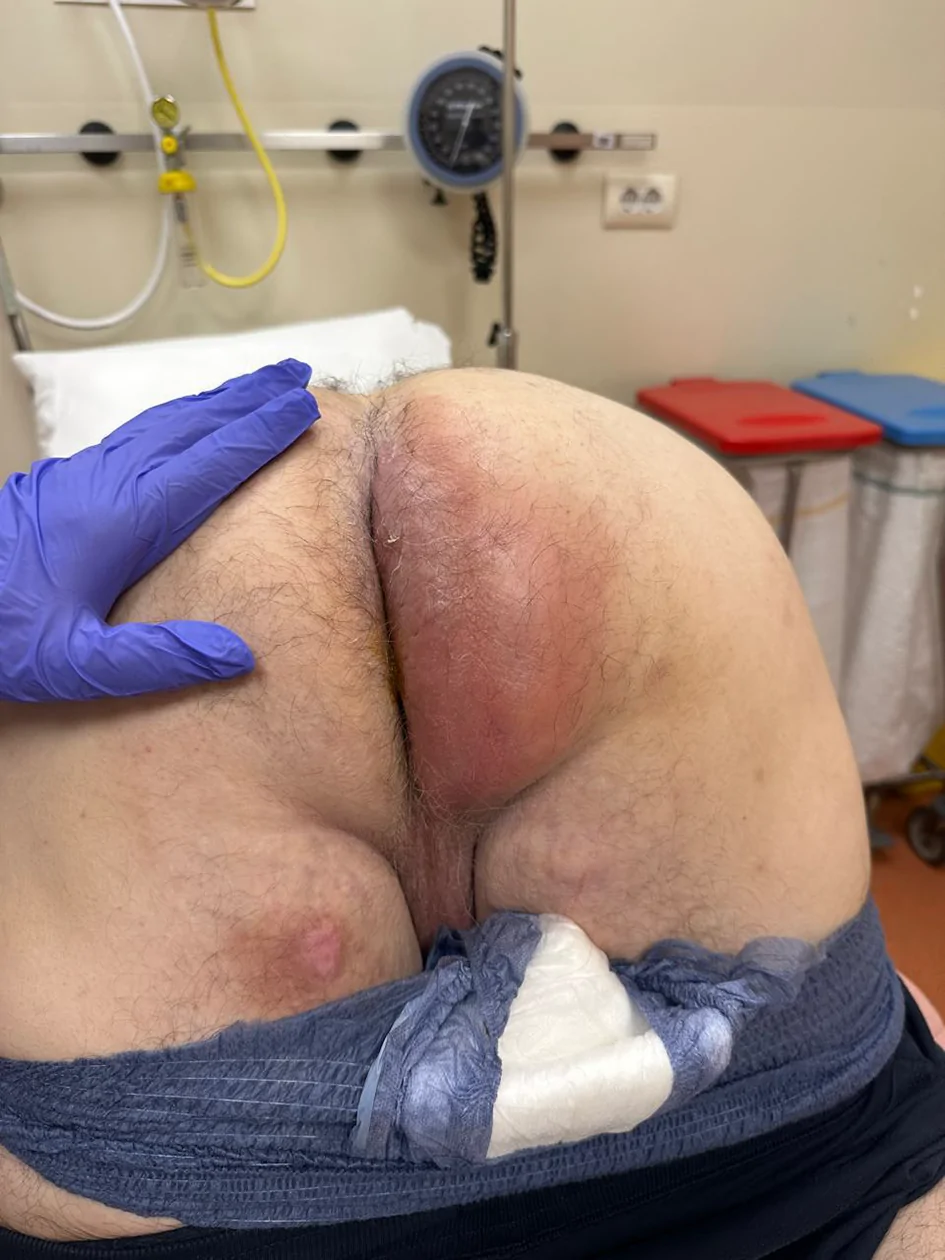
Large perianal abscess.

The patient was obese, with a height of 163 cm and a weight of 112 kg (body mass index (BMI), 42.1 kg/m^2^). His medical history was significant only for well-controlled hypertension managed with chronic anti-hypertensive therapy. Five days before admission, he had received outpatient intravenous antibiotic therapy with clinical improvement. Initial evaluation included routine blood chemistry, a chest radiograph, and surgical consultation. The clinical and laboratory parameters at presentation, together with the corresponding Sequential Organ Failure Assessment (SOFA) components and scores are summarized in Table 1. The total SOFA score was 6 points, indicating acute multisystem organ dysfunction, predominantly involving the respiratory, cardiovascular, and renal systems, in the setting of septic shock.

**Table 1:** Clinical Parameters and Sequential Organ Failure Assessment Score at Admission

Parameter	Patient value	SOFA component	SOFA score
Respiratory rate	26 breaths/min	Respiratory	-
PaO_2_	69 mm Hg	Respiratory	-
Oxygen supplementation	10 L/min Venturi mask	Respiratory	-
Estimated FiO_2_	∼0.40	Respiratory	-
PaO_2_/FiO_2_ ratio	∼173	Respiratory	2
Platelets	170 × 10^3^/µL	Coagulation	0
Bilirubin	1.2 mg/dL	Liver	0
Blood pressure	100/35 mm Hg	Cardiovascular	-
Estimated MAP	∼57 mm Hg	Cardiovascular	-
Norepinephrine	0.05 µg/kg/min	Cardiovascular	3
Heart rate	117 beats/min	-	-
GCS	15	CNS	0
Mental status	Slightly agitated	CNS	-
Creatinine	1.7 mg/dL	Renal	1
Lactate	3.6 mmol/L	-	-
SpO_2_	95%	-	-
Infection	Massive perianal abscess	Clinical context	-
Total SOFA score			6

CNS: central nervous system; GCS: Glascow Comma Scale; MAP: mean arterial pressure; SOFA: Sequential Organ Failure Assessment.

### Diagnosis

Physical examination revealed a giant right perianal abscess extending throughout most of the ipsilateral gluteal region, requiring emergency surgical drainage. Pre-operative chest radiography demonstrated enlargement of the hilar region, and resolving basal pneumonia with oxygen saturation of 95–96% on room air (Fig. 2).

**Figure 2:**
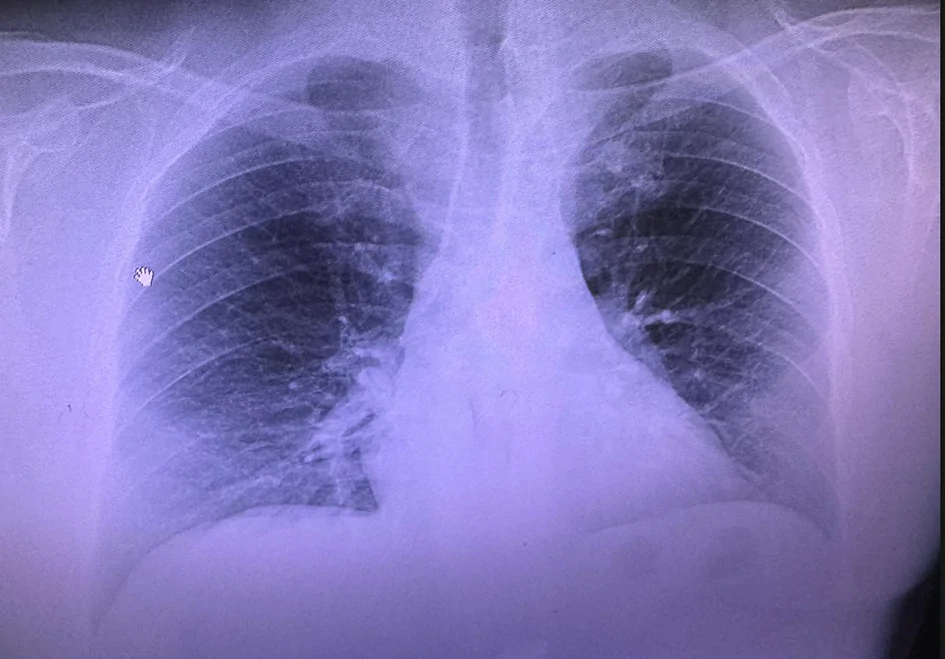
Chest X-ray indicated mild bilateral enlargement of the hilar region, and resolving basal pneumonia.

Transthoracic echocardiography demonstrated preserved LV systolic function with mild-to-moderate MR and no significant regional wall-motion abnormalities.

### Treatment

The patient underwent general anesthesia for drainage of the perianal abscess. The procedure lasted for approximately 1 h and consisted of surgical drainage of the extensive perianal abscess through a large ellipsoid incision made approximately 2 cm from the anal margin, with identification of the internal fistulous opening. Following drainage, a Jackson–Pratt (JP) drainage system was placed, with the drain positioned within the fistulous tract to provide continuous drainage. The drainage system was maintained and managed with regular monitoring and dressing changes for approximately 6 weeks. A trans-sphincteric elastic seton was also placed and subjected to gentle daily traction. Radial arterial cannulation for invasive blood pressure monitoring was performed, in addition to standard monitoring, and general anesthesia was induced with propofol 250 mg, fentanyl 250 µg, and vecuronium 10 mg. Tracheal intubation was uneventful. Anesthesia was maintained with sevoflurane at 1–2% minimum alveolar concentration (MAC) and a remifentanil infusion at 0.3 µg/kg/min. During emergence from anesthesia, the patient developed severe hypertension (201/77 mm Hg; mean arterial pressure (MAP) of 112 mm Hg) accompanied by acute oxygen desaturation, with SpO_2_ ranging from 88% to 92% despite respiratory support in the operating room. Arterial blood gas (ABG) analysis showed a pH of 7.33, PaO_2_ of 51 mm Hg, and PaCO_2_ of 39 mm Hg. Intravenous furosemide (60 mg) was administered, resulting in marked diuresis and progressive improvement in oxygenation. A total of 1,100 mL of normal saline was administered intra-operatively, while urine output reached 1,800 mL following furosemide administration, resulting in a net fluid balance of −700 mL.

Given the acute respiratory deterioration, pulmonary edema was initially suspected. However, the clinical presentation could have resulted from several overlapping factors, including the patient’s previous pneumonia, ongoing sepsis, intra-operative fluid administration, and cardiac dysfunction. A systematic evaluation of these potential causes was therefore undertaken. As the clinical and laboratory findings did not support an alternative explanation for the persistent respiratory impairment, attention increasingly focused on impaired cardiac function as a potential underlying contributor. During the early postoperative period, fever resolved and wound drainage remained satisfactory. However, persistent mild chest discomfort, bilateral basal crackles, and continued oxygen requirements prompted further cardiopulmonary evaluation.

Laboratory investigations demonstrated markedly elevated inflammatory markers, including procalcitonin (2.28 ng/mL), C-reactive protein (27.49 mg/dL), and white blood cell count (25.38 × 10^3^/µL). Surgical-site cultures grew *Staphylococcus epidermidis, Enterococcus faecalis, and Escherichia coli (E. coli)*, while blood culture was positive only for *E. coli*. The patient received a 10-day course of intravenous piperacillin/tazobactam, levofloxacin, and metronidazole. The inflammatory markers, microbiological findings, and antibiotic regimen are summarized in Table 2.

**Table 2:** Inflammatory Markers, Microbiological Findings, and Intravenous Antibiotic Therapy

Parameter	Before treatment	After treatment	Reference range/interpretation
Procalcitonin	2.28 ng/mL	0.41 ng/mL	0.5–2 ng/mL: suspected sepsis; > 2 ng/mL: sepsis
CRP	27.49 mg/dL	0.48 mg/dL	0–0.5 mg/dL
White blood cell count	25.38 × 10^3^/µL	19.45 × 10^3^/µL	4.0–10.5 × 10^3^/µL
Surgical-site microbiological cultures	*Staphylococcus epidermidis; Enterococcus faecalis; Escherichia coli*		
Blood culture	*Escherichia coli*		
Intravenous antibiotic regimen (10 days)	Piperacillin/tazobactam 4.5 g three times daily; levofloxacin 500 mg once daily; metronidazole 500 mg three times daily		

CRP: C-reactive protein.

In contrast to the pre-operative chest radiograph, postoperative chest computed tomography (CT) demonstrated bilateral pleural effusions, pulmonary vascular congestion, diffuse ground-glass opacities, and interlobular septal thickening, findings consistent with pulmonary edema (Fig. 3).

**Figure 3:**
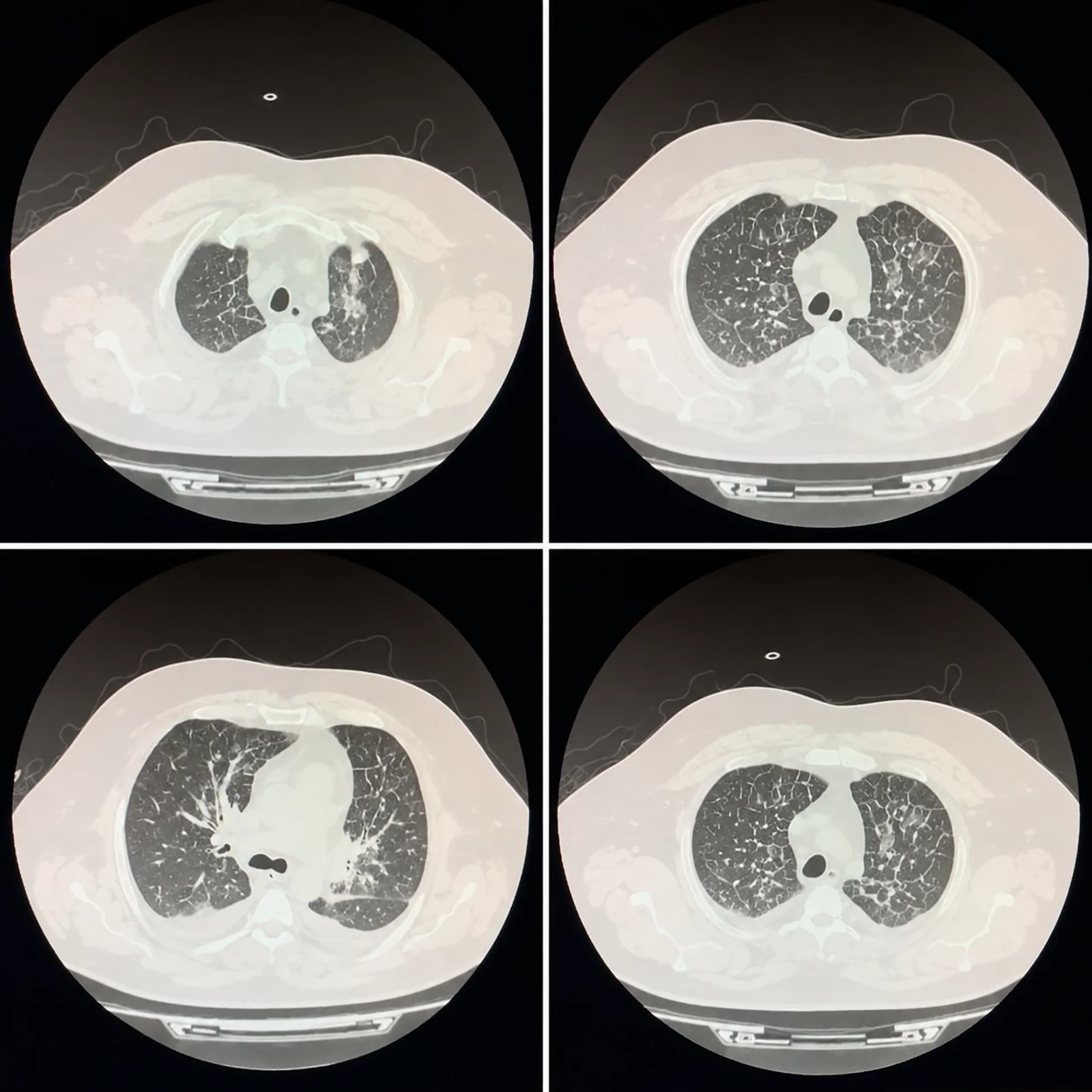
Postoperative chest computed tomography demonstrated bilateral pleural effusions, pulmonary vascular congestion, diffuse ground-glass opacities, and interlobular septal thickening, findings consistent with pulmonary edema.

Following cardiology evaluation, the electrocardiogram (ECG) demonstrated sinus tachycardia at 119 beats/min, with ST-segment depression and T-wave inversion in leads V5 and V6 (Fig. 4). Echocardiography demonstrated a deterioration in cardiac function compared with the pre-operative assessment, with a non-dilated LV showing moderately reduced global systolic function (left ventricle ejection fraction (LVEF) of approximately 35%) and global hypokinesis, without significant regional wall-motion abnormalities. Moderate-to-severe secondary (functional) MR was present, associated with mitral leaflet tethering and LV/mitral annular remodeling. Quantitative parameters included a vena contracta of 5.0 mm, an effective regurgitant orifice area (EROA) of approximately 0.30 cm^2^, and a regurgitant volume of approximately 40 mL/beat. The left atrium was enlarged, with an increased left atrial volume index (LAVI, approximately 38 mL/m^2^). Right-sided chamber enlargement was also noted, with relatively preserved right ventricular (RV) systolic function (tricuspid annular plane systolic excursion (TAPSE): 20 mm; lateral S′: approximately 10 cm/s). The estimated pulmonary artery systolic pressure was approximately 40–45 mm Hg, suggestive of mildly elevated pulmonary pressure, provided the measurement was technically reliable. Mild tricuspid regurgitation was present, with no significant pericardial effusion. Cardiac biomarkers were markedly elevated, with high-sensitivity troponin of 1,950 ng/L (normal range: 0–0.019 ng/L) and creatine kinase myocardial band (CK-MB) of 350 ng/mL (normal range: < 6.22 ng/mL), raising suspicion of acute myocardial infarction. In the immediate postoperative period, due to reduced cardiac output, the patient developed hemodynamic compromise characterized by hypotension (95/33 mm Hg; MAP: 55 mm Hg), an increase in serum lactate to 7.1 mmol/L, central venous oxygen saturation (ScvO_2_) of 66%, and prolonged capillary filling. Given the evidence of acute myocardial dysfunction with impaired perfusion despite conservative fluid management, dobutamine was initiated for inotropic support at 7.5 µg/kg/min, while norepinephrine was administered to support arterial pressure at 0.05–0.1 µg/kg/min. These doses represented the maximum infusion rates used. Hemodynamic status, tissue perfusion, and serial lactate levels were closely monitored. With progressive clinical improvement, normalization of tissue perfusion and lactate levels, and recovery of hemodynamic stability, dobutamine and norepinephrine were progressively tapered and discontinued on approximately the fourth to fifth postoperative day. The patient underwent urgent coronary angiography, which revealed only a non-obstructive 40% stenosis of the right coronary artery, normal left coronary artery tree, with no indication for percutaneous coronary stents insertion (Fig. 5). CMR was performed on postoperative day 3 using a Siemens 1.5-T system and included standard cine imaging, native T1 and T2 mapping, and late gadolinium enhancement (LGE) imaging following intravenous administration of a gadolinium-based contrast agent at 0.1 mmol/kg, followed by a saline flush. The LV showed global systolic dysfunction, with diffuse hypokinesia and a CMR-derived LVEF of 35%. RV systolic function was relatively preserved, with a right ventricle ejection fraction (RVEF) of 50%. Native T1 values were elevated in the basal-to-mid-inferolateral and anterolateral LV segments, ranging from 1,080 to 1,100 ms (institutional reference range: 950–1,050 ms). T2 mapping demonstrated corresponding regional elevation, ranging from 57 to 64 ms (institutional reference range: 44–58 ms), consistent with active myocardial edema. The extracellular volume (ECV) fraction was mildly increased at 28% (institutional reference value: < 25%). LGE imaging demonstrated patchy subepicardial to mid-myocardial enhancement predominantly involving the basal and mid-inferolateral and anterolateral LV segments, with a characteristic non-ischemic distribution. During LGE assessment, the LV mass was approximately 14% higher than normal. No subendocardial or transmural LGE pattern suggestive of myocardial infarction was identified.

**Figure 4:**
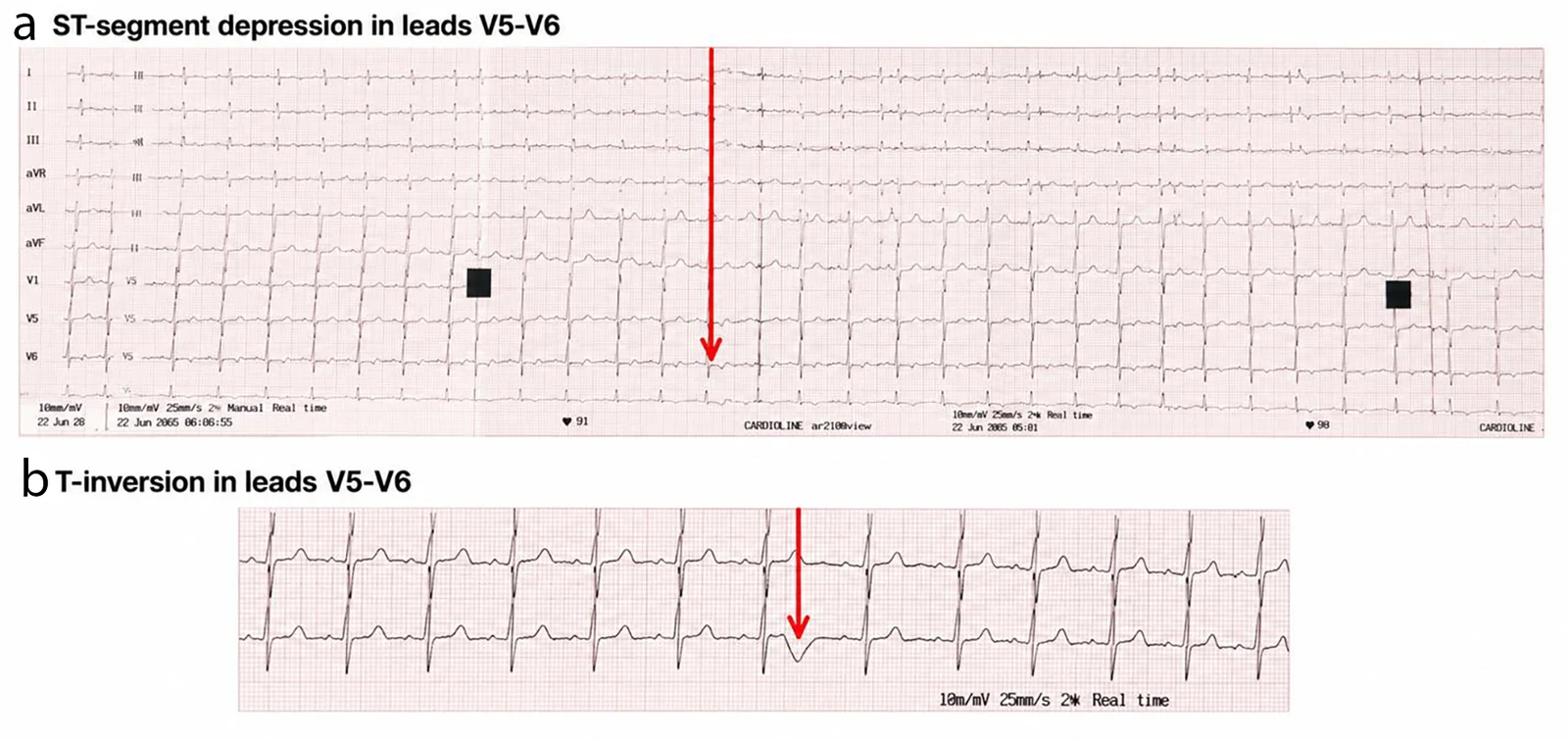
ECG indicated tachycardia, atrial extrasystoles, ST-segment depression and T-inversion in leads V5–V6. ECG: electrocardiogram.

**Figure 5:**
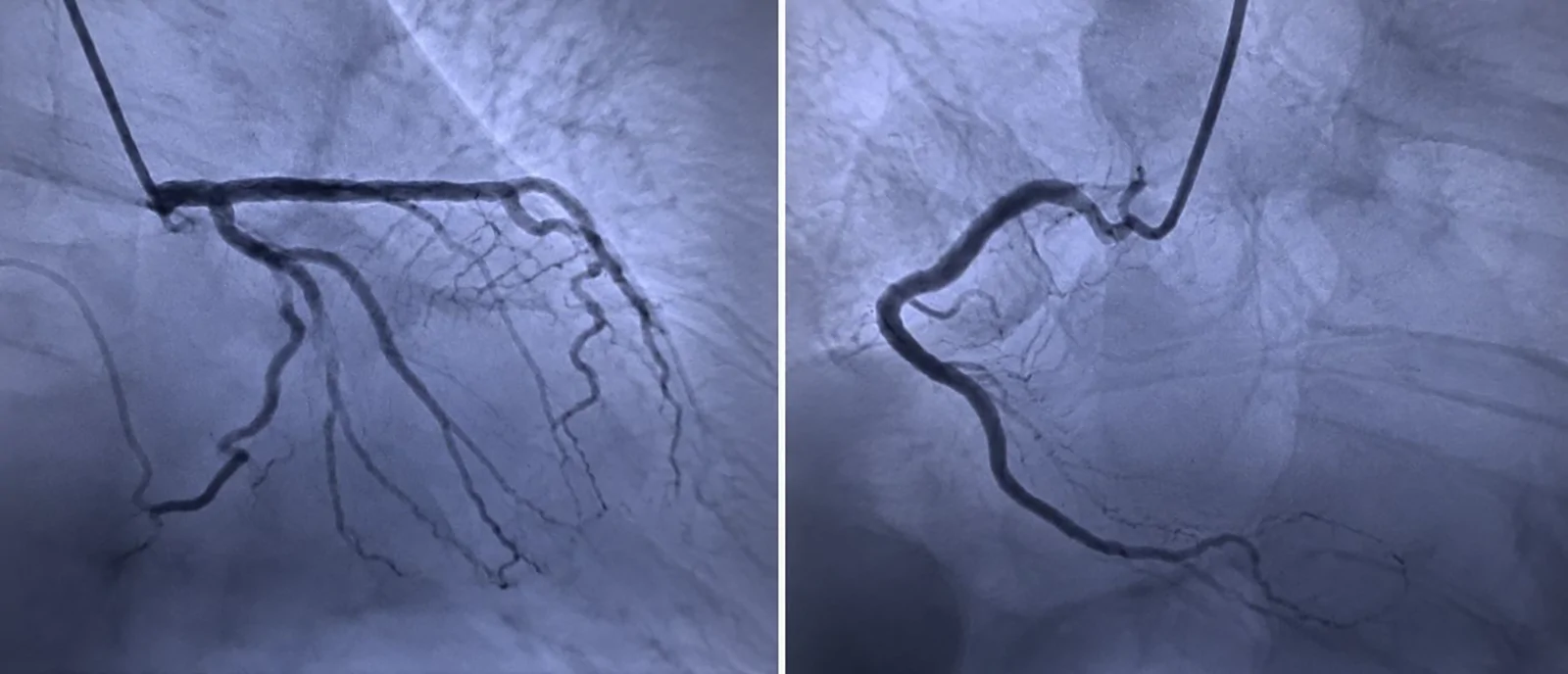
Urgent coronary angiography demonstrated a non-obstructive 40% stenosis of the right coronary artery, while the left coronary arterial system was angiographically normal.

The combination of positive T1 and T2-based tissue characterization criteria fulfilled the 2018 revised Lake Louise Criteria, supporting the diagnosis of acute myocarditis. Moderate-to-severe MR was also present, with an estimated regurgitant fraction of 35–40%. No significant pericardial effusion was observed. CMR demonstrated acute myocarditis, characterized by active myocardial edema and non-ischemic myocardial injury, associated with significant global LV systolic dysfunction (LVEF: 35%) and an approximately 14% increase in LV myocardial mass, a finding considered supportive of acute myocardial inflammation when exceeding the reported 10–15% threshold. No evidence of myocardial infarction was identified (Fig. 6).

**Figure 6:**
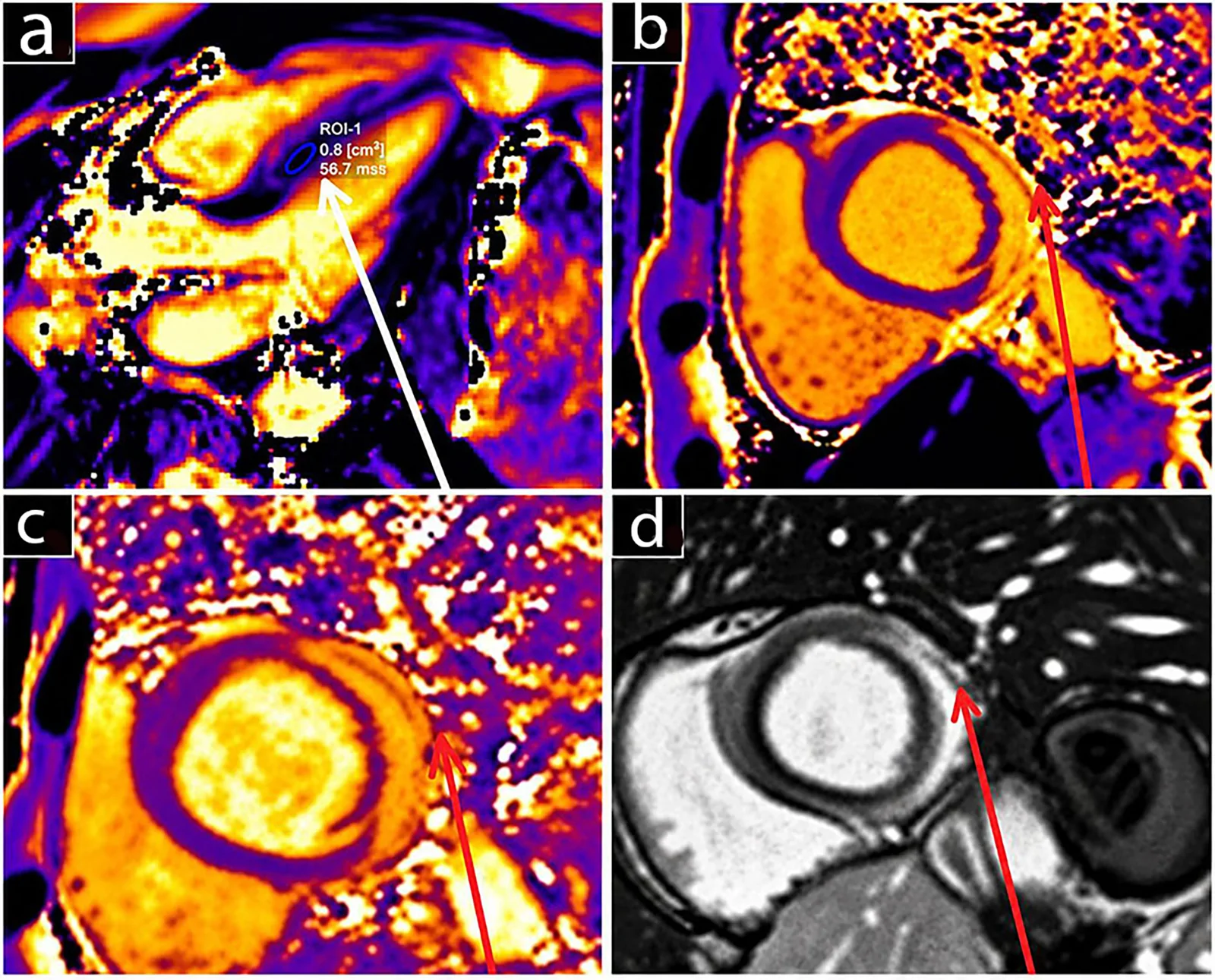
Cardiac magnetic resonance imaging indicated: (a) Positive T2 map in septal region with LGE. (b) Positive T1 map in lateral wall region with LGE. (c) Positive T2 map in lateral wall region with LGE. (d) Late gadolinium enhancement. Non-ischemic epicardial in the lateral wall. LGE: Late gadolinium enhancement.

The combination of markedly elevated cardiac biomarkers, non-obstructive coronary arteries, septic presentation, and subsequent clinical course initially raised suspicion for ACS, including non-obstructive coronary arteries (MINOCA). However, CMR with tissue characterization performed after stabilization supported acute myocarditis as the more likely non-ischemic diagnosis. The stepwise diagnostic evaluation, including the exclusion of alternative causes and the findings supporting the final diagnosis, is summarized in Table 3.

**Table 3:** Step-by-Step Diagnostic Evaluation and Establishment of the Final Diagnosis

Step	Diagnostic question/challenge	Examination/finding	Diagnostic conclusion/exclusion
1. Respiratory deterioration	What caused the acute postoperative hypoxemia?	ABG showed PaO_2_ of 51 mm Hg; chest CT showed pulmonary vascular congestion, bilateral pleural effusions, ground-glass opacities, and septal thickening	Pulmonary edema identified
2. Fluid overload	Could pulmonary edema be explained by excessive fluid administration?	1,100 mL intra-operative fluids; 1,800 mL urine output after furosemide; net fluid balance −700 mL	Simple fluid overload was less likely
3. New cardiac dysfunction	Was there acute deterioration in cardiac function?	Echocardiography: LVEF ∼35%, global hypokinesis, new moderate-to-severe functional MR, without significant regional wall-motion abnormalities	Acute myocardial dysfunction confirmed, with a predominantly global rather than regional pattern
4. Acute myocardial injury	Was the cardiac deterioration associated with myocardial injury?	Hs-troponin 1,950 ng/L and CK-MB 350 ng/mL; ECG showed ST depression and T-wave inversion in V5–V6	ACS was strongly suspected
5. Obstructive coronary disease	Was this an acute myocardial infarction due to coronary obstruction?	Urgent coronary angiography: only 40% non-obstructive RCA stenosis; normal left coronary system	Obstructive ACS excluded; MINOCA considered
6. Myocarditis vs. septic cardiomyopathy	What explained the myocardial injury in the setting of severe sepsis?	CMR: elevated T2 values indicating myocardial edema and LGE involving the interventricular septum and LV lateral wall	Active myocardial inflammation demonstrated, supporting acute myocarditis
7. Histological confirmation	Could myocarditis be confirmed by biopsy?	Endomyocardial biopsy was declined by the patient	Diagnosis was established non-invasively by clinical context, exclusion of obstructive CAD, and CMR findings
8. Diagnostic confirmation by outcome	Was the myocardial dysfunction reversible?	LVEF recovered to 55%, MR resolved, NT-proBNP decreased from 3,846 to 95 pg/mL, and cardiac enzymes normalized	Reversible inflammatory myocardial injury consistent with acute myocarditis

ABG: arterial blood gas; ACS: acute coronary syndrome; CAD: coronary artery disease; CMR: cardiac magnetic resonance; CT: computed tomography; CK-MB: creatine kinase myocardial band; ECG: electrocardiogram; MINOCA: myocardial infarction with non-obstructive coronary arteries; MR: mitral regurgitation; LGE: late gadolinium enhancement; LV: left ventricular; LVEF: left ventricle ejection fraction; NT-proBNP: N-terminal pro-B-type natriuretic peptide; RCA: right coronary artery.

The clinical course from symptom onset through hospital admission, surgery, postoperative deterioration, cardiac evaluation, treatment, and follow-up is summarized chronologically in Table 4.

**Table 4:** Clinical Timeline of Symptom Onset, Peri-Operative Deterioration, Diagnostic Evaluation, Treatment, and Follow-Up

Time point	Clinical course/symptoms	Investigations and findings	Treatment/management	Outcome / diagnostic interpretation
Day 10	Fever (40 °C), right perianal pain/swelling, mild cough, and fatigue	Large perianal abscess identified	-	Severe local infection with systemic inflammatory response.
Day 5	Persistent symptoms with temporary improvement	-	Outpatient IV antibiotics	Subsequent clinical deterioration.
Day 0 – hospital admission	Fever 40 °C, tachypnea, diaphoresis; BMI 42.1 kg/m^2^	SOFA score 6; large perianal abscess	Broad-spectrum antibiotics; surgical consultation	Severe sepsis/septic shock secondary to perianal abscess.
Day 0 – pre-operative assessment	Large right perianal abscess extending into the gluteal region	CXR: hilar enlargement/resolving pneumonia; SpO_2_ 95–96%. Echo: preserved LV function, mild-to-moderate MR	Emergency surgical drainage planned	Normal LV systolic baseline.
Day 0 – surgery	Emergency abscess drainage	Radial arterial monitoring; uncomplicated intubation	General anesthesia; propofol, fentanyl, vecuronium, sevoflurane, remifentanil	Surgery completed in ∼1 h.
Day 0 – emergence	Severe hypertension (201/77 mm Hg) and hypoxemia (SpO_2_ 88–92%)	ABG: pH 7.33, PaO_2_ 51, PaCO_2_ 39 mm Hg	Furosemide 60 mg IV; net fluid balance −700 mL	Pulmonary edema initially suspected.
Early postoperative period	Persistent oxygen requirement, chest discomfort, and basal crackles	CT: bilateral pleural effusions, vascular congestion, ground-glass opacities, septal thickening	Cardiopulmonary monitoring and cardiac evaluation	Cardiac dysfunction increasingly suspected.
Early postoperative period – cardiac evaluation	New cardiac dysfunction	ECG: sinus tachycardia 119 bpm, ST depression/T-wave inversion V5–V6. Echo: LVEF 35%, moderate to severe MR. Hs-Tn 1,950 ng/L; CK-MB 350 ng/mL	Cardiology consultation	ACS/acute MI initially suspected.
Early postoperative ICU period	Hemodynamic instability requiring intensive monitoring	Adequate tissue perfusion maintained	Dobutamine 7.5 µg/kg/min + norepinephrine 0.05–0.1 µg/kg/min	Hemodynamic stabilization.
Early ICU period – coronary evaluation	Ongoing myocardial injury evaluation	40% non-obstructive RCA stenosis; normal left coronary tree	No coronary intervention	Obstructive ACS excluded; MINOCA/non-ischemic injury considered.
After hemodynamic stabilization – CMR	Persistent concern for myocarditis	CMR: elevated septal T2 values and LGE involving the interventricular septum/LV lateral wall	EMB declined	Findings supported acute myocarditis.
ICU days 1–6	Progressive cardiac and respiratory improvement	Stable tissue perfusion	Antibiotics, diuretics, oxygen, vasoactive/inotropic support	Transferred to cardiology after 6 days.
Day 6 – transfer from ICU	Continued improvement	-	Diuretics, ACE inhibitor, digitalis, oxygen, antibiotics, wound care	Continued recovery.
Day 10	Ongoing recovery	Echo: LVEF 45%, moderate residual MR	Conservative heart failure management	Improved LV function.
Three-month follow-up	Clinically recovered	Complete recovery of cardiac function; MR resolved	Follow-up	Complete healing of perianal wound/fistula and favorable outcome.

ABG: arterial blood gas; ACE: angiotensin converting enzyme; BMI: body mass index; CT: computed tomography; CMR: cardiac magnetic resonance; CXR: chest X-ray; CK-MB: creatine kinase myocardial band; ECG: electrocardiogram; EMB: endomyocardial biopsy; hs-Tn: hs-troponin; ICU: intensive care unit; IV: intravenous; LGE: late gadolinium enhancement; LV: left ventricular; LVEF: left ventricle ejection fraction; MR: mitral regurgitation; RCA: right coronary artery; SOFA: Sequential Organ Failure Assessment.

### Follow-up and outcomes

After 6 days of intensive care unit (ICU) treatment, the patient was transferred to the cardiology department and managed conservatively with intravenous diuretics, an angiotensin-converting enzyme inhibitor, oxygen therapy, antibiotics, and ongoing surgical wound care.

The patient’s clinical condition improved progressively. Repeat transthoracic echocardiography performed 10 days later demonstrated an LVEF of 45% and moderate residual MR.

At the 3-month follow-up, transthoracic echocardiography demonstrated further recovery of LV systolic function, with an LVEF of 55% and complete resolution of MR. LV end-systolic and end-diastolic dimensions were within normal limits. The N-terminal pro-B-type natriuretic peptide (NT-proBNP) level had markedly decreased from 3,846 pg/mL at presentation (reference value: < 87 pg/mL) to 95 pg/mL, while cardiac enzyme levels were within normal limits. The patient was clinically stable and classified as New York Heart Association (NYHA) functional class II. No adverse events or rehospitalizations occurred during follow-up. Holter monitoring and exercise testing were not performed. At the 3-month follow-up, the patient was receiving ramipril 5 mg once daily, dapagliflozin 10 mg once daily, eplerenone 25 mg once daily, furosemide 40 mg once daily, and bisoprolol 5 mg once daily. Clinical examination documented complete healing of the perianal wound and secondary fistula. The wound was managed with ongoing local care and follow-up until progressive healing was achieved. At the 3-month follow-up, complete healing of the perianal wound and secondary fistula was clinically documented.

## Discussion

SICM is a heterogeneous and potentially reversible form of myocardial dysfunction that develops during severe infection. Its reported incidence varies considerably because of differences in diagnostic definitions, although contemporary data suggest that approximately one-third of patients with septic shock develop some degree of myocardial dysfunction. SICM is associated with greater hemodynamic instability, prolonged intensive care requirements, and increased mortality [7, 8]. Its pathogenesis involves dysregulated inflammation, endothelial and microcirculatory dysfunction, oxidative stress, mitochondrial injury, altered calcium homeostasis, and impaired β-adrenergic signaling. These mechanisms may result in transient ventricular dysfunction without requiring direct myocardial infection [9, 10].

In the present case, however, the principal diagnostic challenge was not simply recognition of myocardial dysfunction during sepsis but determination of its cause. Severe bacterial sepsis secondary to an extensive perineal abscess was followed by marked cardiac biomarker elevation, dynamic electrocardiographic abnormalities, reduced LV systolic function, and moderate-to-severe MR. Because these findings could represent ACS, SICM, or myocarditis, they could not initially be attributed to sepsis-induced myocardial dysfunction alone. Echocardiography remains central to the initial assessment of myocardial dysfunction, whereas CMR can provide additional tissue characterization when the diagnosis remains uncertain [11].

Sato et al describe SICM as a heterogeneous syndrome encompassing different patterns of ventricular dysfunction rather than a single disease phenotype. Consequently, treatment is directed primarily toward control of sepsis and individualized hemodynamic support according to the patient’s cardiovascular phenotype [12]. Rapid source control, appropriate antimicrobial therapy, individualized fluid management, and vasoactive or inotropic support when indicated remain the principal therapeutic strategies, as no specific disease-directed treatment has yet demonstrated a consistent survival benefit [13, 14].

The relationship between sepsis and myocarditis is particularly important in this context. Severe sepsis may produce myocardial injury through systemic inflammatory and microvascular mechanisms, creating substantial overlap with acute myocarditis. Distinguishing the two is difficult because both may present with elevated cardiac biomarkers, reduced LV systolic function, ventricular dilatation, and hemodynamic instability. In the present patient, the degree and pattern of myocardial injury, together with dynamic ECG abnormalities and the subsequent course, prompted further evaluation rather than attribution of the cardiac findings to sepsis alone [15]. The possibility of acute myocarditis should therefore be considered when myocardial dysfunction appears disproportionate to the severity of septic shock or persists despite adequate source control and circulatory resuscitation [16]. The key clinical and diagnostic features that may help distinguish SICM from myocarditis are summarized in Table 5 [17].

**Table 5:** Differential Diagnosis Between Sepsis-Induced Cardiomyopathy and Myocarditis

Feature	Sepsis-induced cardiomyopathy	Myocarditis
Underlying cause	Systemic inflammatory response to sepsis causing reversible myocardial dysfunction	Inflammatory injury of the myocardium, most commonly viral, autoimmune, toxic, or immune-mediated
Clinical context	Occurs during severe sepsis or septic shock	May occur following a viral prodrome, autoimmune disease, drug exposure, or independently of sepsis
Typical onset	Concurrent with septic illness	Acute, subacute, or chronic; may precede or occur without systemic infection
Symptoms	Signs of sepsis with circulatory failure; heart failure symptoms may be masked	Chest pain, dyspnea, palpitations, fatigue, syncope, heart failure
Fever	Common due to sepsis	May be present if infectious; absent in autoimmune forms
Hemodynamics	Vasodilatory shock with reduced systemic vascular resistance; myocardial depression contributes to hypotension	Variable; may range from stable hemodynamics to cardiogenic shock (fulminant myocarditis)
ECG findings	Sinus tachycardia; non-specific ST-T changes; occasional arrhythmias	ST-segment and T-wave abnormalities, PR depression, conduction disturbances, ventricular arrhythmias
Cardiac biomarkers	Troponin mildly to moderately elevated; BNP/NT-proBNP frequently elevated	Troponin often markedly elevated; BNP/NT-proBNP elevated with ventricular dysfunction
Inflammatory markers	Markedly elevated (CRP, procalcitonin, leukocytosis) reflecting sepsis	Elevated CRP and ESR; procalcitonin usually normal unless concomitant bacterial infection
Echocardiography	Global biventricular systolic dysfunction, ventricular dilatation, reduced ejection fraction, usually without regional wall motion abnormalities	Regional or global ventricular dysfunction; increased wall thickness (edema), pericardial effusion may be present
Speckle-tracking echocardiography	Reduced global longitudinal strain, usually diffuse	Reduced GLS with regional abnormalities corresponding to inflamed myocardium
Cardiac magnetic resonance	Usually non-specific; myocardial edema may occur, but late gadolinium enhancement is uncommon	Diagnostic modality of choice; demonstrates myocardial edema and non-ischemic LGE according to updated Lake Louise criteria
Coronary angiography	Usually normal if performed	Usually normal unless concomitant coronary artery disease
Endomyocardial biopsy	Not indicated	Gold standard for definitive diagnosis in selected patients, particularly fulminant or unexplained cases
Treatment	Source control, antibiotics, hemodynamic support, vasopressors/inotropes, management of septic shock	Guideline-directed heart failure therapy, treatment of underlying cause, immunosuppression in selected cases, mechanical circulatory support if required
Recovery	Usually reversible within 7–10 days after resolution of sepsis	Variable; complete recovery, persistent ventricular dysfunction, dilated cardiomyopathy, or death
Prognosis	Primarily determined by severity of sepsis rather than cardiac dysfunction alone	Depends on etiology and severity; fulminant myocarditis has high early mortality, but survivors often recover well

BNP: B-type natriuretic peptide; CRP: C-reactive protein; ECG: electrocardiogram; ESR: erythrocyte sedimentation rate; GLS: global longitudinal strain; LGE: late gadolinium enhancement; NT-proBNP: N-terminal pro-B-type natriuretic peptide.

Acute myocarditis has a broad clinical spectrum, ranging from mild or subclinical myocardial injury to acute heart failure, malignant ventricular arrhythmias, cardiogenic shock, and sudden cardiac death. Its presentation may closely mimic ACS, particularly when chest pain or ECG abnormalities are accompanied by marked troponin elevation and ventricular dysfunction. The estimated annual incidence is approximately 10–25 cases per 100,000 population, although the true burden is probably underestimated because many cases remain unrecognized. The disease predominantly affects younger adults, particularly men, and may result in persistent ventricular dysfunction or inflammatory cardiomyopathy [18, 19].

The etiology of myocarditis is heterogeneous and includes infectious and non-infectious mechanisms. Viral infections remain common, but bacterial and other infectious causes, autoimmune and hypersensitivity disorders, toxins, drugs, and genetic predisposition should also be considered. The Spanish Society of Cardiology consensus emphasizes that myocarditis represents a heterogeneous inflammatory syndrome and that identifying the underlying mechanism is important because prognosis and treatment may vary according to etiology [20, 21].

Several clinical features are associated with increased risk and adverse outcome, including reduced LVEF, ventricular arrhythmias, advanced conduction abnormalities, cardiogenic shock, persistent biomarker elevation, and extensive myocardial fibrosis on CMR. Patients with such features require close monitoring and, when necessary, escalation to advanced circulatory support [22].

In the present patient, the diagnostic process was sequential. Marked troponin elevation, ST-segment depression with T-wave inversion in the lateral leads, and newly reduced LV systolic function initially raised strong suspicion for acute myocardial infarction. Because myocardial injury in a septic patient should not automatically be attributed to SICM, urgent coronary angiography was performed. The demonstration of only mild non-obstructive coronary disease substantially reduced the likelihood of an acute obstructive coronary event and redirected the diagnostic evaluation toward alternative causes of myocardial injury. The subsequent diagnostic assessment integrated the clinical presentation, biomarkers, ECG, echocardiography, coronary angiography, and CMR. CMR demonstrated myocardial edema on T2 mapping together with LGE involving the interventricular septum, providing evidence of active myocardial inflammation and supporting the diagnosis of acute myocarditis. EMB remains the diagnostic reference standard in selected high-risk presentations, but current recommendations reserve biopsy for situations in which histological or molecular confirmation is expected to influence management [23, 24].

Management of acute myocarditis is primarily supportive and depends on disease severity and the suspected underlying cause. Patients with ventricular dysfunction require appropriate heart failure management and monitoring for arrhythmias, while fulminant presentations may require vasoactive or inotropic support and temporary mechanical circulatory support. Etiology-specific immunosuppressive therapy is reserved for selected forms after infectious causes have been excluded. Follow-up is important because persistent ventricular dysfunction or arrhythmias may occur despite apparent clinical recovery [2, 25]. The principal diagnostic elements of myocarditis are summarized in Table 6 [26].

**Table 6:** Key Diagnostic Elements for Myocarditis

Diagnostic modality	Key findings	Diagnostic value
Clinical presentation	Chest pain, dyspnea, palpitations, syncope, fatigue, acute or chronic heart failure; recent viral illness or autoimmune disease may be present	Raises clinical suspicion but is not diagnostic
Electrocardiogram	Sinus tachycardia, ST-segment elevation or depression, T-wave inversion, PR depression, AV block, bundle branch block, ventricular arrhythmias	Non-specific; useful for identifying conduction abnormalities and arrhythmias
Cardiac biomarkers	Elevated cardiac troponin (I or T); elevated CK-MB; elevated BNP or NT-proBNP if ventricular dysfunction is present	Indicates myocardial injury but does not establish the cause
Inflammatory markers	Elevated C-reactive protein, erythrocyte sedimentation rate, leukocytosis; procalcitonin may help identify concomitant bacterial infection	Supports inflammatory process but lacks specificity
Transthoracic echocardiography	Regional or global left ventricular dysfunction, reduced ejection fraction, increased wall thickness due to edema, diastolic dysfunction, pericardial effusion	First-line imaging to assess cardiac function and exclude alternative diagnoses
Speckle-tracking echocardiography	Reduced global longitudinal strain, often with regional impairment	Detects subtle myocardial dysfunction before EF declines
Cardiac magnetic resonance	Myocardial edema (T2-weighted imaging or T2 mapping), non-ischemic late gadolinium enhancement, increased native T1 and extracellular volume	Preferred non-invasive diagnostic test; diagnosis based on the updated Lake Louise Criteria
Coronary angiography or CT coronary angiography	Normal coronary arteries	Excludes acute coronary syndrome in appropriate patients
Endomyocardial biopsy	Histological evidence of inflammatory infiltrates with myocyte necrosis; immunohistochemistry and molecular testing can identify inflammatory cell type and viral genome	Gold standard for definitive diagnosis; recommended in selected patients (e.g., fulminant, unexplained, or treatment-resistant myocarditis)
Virological and autoimmune testing	Viral PCR, serology (limited role), autoimmune antibodies, eosinophil count when clinically indicated	Identifies underlying etiology and guides management in selected cases
Diagnostic criteria	Combination of compatible clinical presentation with CMR findings and/or histological confirmation by EMB	Diagnosis should integrate clinical, imaging, laboratory, and histopathological findings

AV: atrioventricular; BNP: B-type natriuretic peptide; CK-MB: creatine kinase myocardial band; CMR: cardiac magnetic resonance; CT: computed tomography; EF: ejection fraction; EMB: endomyocardial biopsy; NT-proBNP: N-terminal pro-B-type natriuretic peptide; PCR: polymerase chain reaction.

In this case, the CMR findings provided the strongest evidence supporting acute myocarditis, but they should be interpreted in the context of severe bacterial sepsis. The absence of significant obstructive coronary disease and the presence of myocardial edema and non-ischemic LGE favored inflammatory myocardial injury rather than acute coronary occlusion. Nevertheless, SICM cannot be completely excluded. SICM and myocarditis may share clinical, biochemical, and echocardiographic features, and inflammatory myocardial injury may occur alongside sepsis-associated myocardial dysfunction. Therefore, the diagnosis in this patient is best regarded as CMR-supported acute myocarditis occurring in the setting of severe bacterial sepsis, with possible overlap with SICM.

The peri-operative course further illustrates the diagnostic challenge. At the time of emergency surgery, myocarditis had not yet been diagnosed, and the immediate priority was source control of severe infection. Consequently, the anesthetic strategy was directed toward maintaining cardiovascular stability in a septic patient with potentially limited myocardial reserve rather than toward management of an established diagnosis of myocarditis. The principal considerations were careful hemodynamic assessment, avoidance of abrupt changes in preload and afterload, and minimization of myocardial depression during anesthesia [27].

Pre-operative assessment was necessarily limited by the urgency of surgery. Nevertheless, available echocardiographic information and hemodynamic status should guide peri-operative management in patients with suspected or established ventricular dysfunction. Invasive arterial pressure monitoring may facilitate early recognition and treatment of hemodynamic deterioration during major emergency procedures [28]. Anesthetic drugs should be carefully titrated, with attention to preservation of preload, sinus rhythm, and stable systemic vascular resistance [29]. Excessive cardiovascular depression should be avoided, and drug selection should be individualized according to the patient’s hemodynamic condition [30].

During emergence from anesthesia, the patient developed acute pulmonary edema with severe hypertension and hypoxemia, which responded to intravenous furosemide and aggressive diuresis. Subsequent intensive care management included invasive hemodynamic monitoring, norepinephrine and dobutamine, antimicrobial therapy, optimization of tissue perfusion, and close cardiopulmonary surveillance. The need for coordinated management among anesthesiology, intensive care, cardiology, radiology, and surgery was particularly important because the myocardial diagnosis evolved after the emergency operation rather than being established pre-operatively [31].

The subsequent clinical course was favorable, with progressive improvement in LV systolic function, complete resolution of MR, and normalization of cardiac function at 3 months without coronary intervention. This course is compatible with reversible inflammatory myocardial injury. However, recovery alone cannot distinguish myocarditis from SICM because both conditions may improve following resolution of severe infection. The diagnostic strength of this case therefore rests on the combination of marked myocardial injury, dynamic ECG abnormalities, exclusion of significant obstructive coronary disease, and characteristic CMR findings rather than on recovery alone.

The case also emphasizes that the anatomical source of infection, although unusual, is less important than the diagnostic reasoning and transferable clinical lesson. In septic patients with unexpected or disproportionate myocardial injury, marked troponin elevation, dynamic ECG abnormalities, new ventricular dysfunction, or pulmonary edema should not automatically be attributed to SICM. ACS should initially be considered and excluded when clinically indicated. When significant coronary obstruction is absent, myocarditis and other causes of myocardial injury should remain in the differential diagnosis, and CMR can provide decisive non-invasive tissue characterization. In the present case, this sequential approach allowed acute coronary occlusion to be excluded and myocarditis to be supported by CMR (Table 7).

**Table 7:** Comparison of the Present Case With the Clinically Relevant Evidence

Reference	Focus of the cited study/recommendation	Relevance to the present case	Present case: corresponding finding	Contribution to diagnostic reasoning
Beesley et al [4]	Sepsis-induced cardiomyopathy and its clinical recognition	Establishes SICM as an important alternative explanation for myocardial dysfunction during sepsis	New global LV dysfunction developed during severe bacterial sepsis	SICM had to be considered before attributing the dysfunction to myocarditis
Romero-Bermejo et al [7]	Sepsis-induced cardiomyopathy	Describes reversible myocardial dysfunction associated with sepsis	LVEF decreased from preserved pre-operatively to 35% postoperatively	Demonstrates why septic cardiomyopathy was an important differential diagnosis
Borkowski et al [8]	Clinical complexity of sepsis-induced cardiomyopathy	Highlights diagnostic overlap between sepsis and cardiac dysfunction	Marked troponin elevation, LV dysfunction and hemodynamic instability occurred in the setting of sepsis	Supports the need for systematic cardiac evaluation rather than assuming SICM
Aissaoui et al [9]	Contemporary understanding, diagnosis, and assessment of SICM	Provides the framework for evaluating myocardial dysfunction in sepsis	Echocardiography demonstrated new global LV dysfunction without significant regional abnormalities	Global dysfunction supported SICM as a differential but did not establish it
Sato et al [12]	SICM as a heterogeneous syndrome and role of echocardiography	Emphasizes multimodal assessment of septic cardiac dysfunction	Serial echocardiography demonstrated marked deterioration followed by recovery	Serial cardiac imaging helped establish the evolution and reversibility of dysfunction
Martin et al [15]	Mechanisms and clinical implications of the septic heart	Explains inflammatory myocardial injury during severe sepsis	Severe bacterial sepsis coincided with marked myocardial injury	Provides biological plausibility for sepsis-associated inflammatory myocardial injury
Lampejo et al [18]	Diagnosis and management of acute myocarditis	Defines the clinical and diagnostic spectrum of myocarditis	Troponin elevation, ECG abnormalities, and new LV dysfunction	Demonstrates that the patient’s presentation was compatible with acute myocarditis but non-specific
Ammirati and Moslehi [19]	Diagnosis and treatment of acute myocarditis	Supports multimodal diagnostic assessment	CMR was performed after coronary angiography excluded obstructive CAD	Supports the stepwise transition from suspected ACS to myocarditis
Domínguez et al [20]	Consensus approach to myocarditis and inflammatory cardiomyopathy	Supports integration of clinical, laboratory, and imaging findings	Clinical context + biomarkers + echocardiography + coronary angiography + CMR	Reinforces the multimodal diagnostic approach used in this case
Ferreira et al [26]	CMR evaluation of non-ischemic myocardial inflammation	Directly relevant to the decisive investigation	Elevated T2 values and non-ischemic LGE in the septum/LV lateral wall	CMR provided the principal non-invasive evidence supporting acute myocarditis

ACS: acute coronary syndrome; CAD: coronary artery disease; CMR: cardiac magnetic resonance; ECG: electrocardiogram; LGE: late gadolinium enhancement; LV: left ventricular; LVEF: left ventricle ejection fraction; SICM: sepsis-induced cardiomyopathy.

Several limitations should be acknowledged. First, EMB was declined by the patient; therefore, histopathological confirmation of myocardial inflammation and molecular identification of a causative pathogen were unavailable. Second, although CMR provided important evidence supporting myocarditis, it could not establish with certainty the precise etiology of the myocardial inflammation or prove a direct causal relationship with the bacterial infection. Third, concomitant SICM cannot be completely excluded because septic myocardial dysfunction and myocarditis may overlap clinically and pathophysiologically. Finally, complete baseline cardiac characterization was unavailable, limiting certainty regarding the patient’s pre-existing ventricular function and whether any cardiac abnormalities preceded the acute illness.

This case highlights the importance of maintaining diagnostic flexibility when significant myocardial injury develops during sepsis. The practical lesson is that myocardial dysfunction in a septic patient should not automatically be labeled as SICM, particularly when the degree or pattern of injury is unexpected. A structured diagnostic pathway, recognition of myocardial injury, assessment for ACS, coronary angiography when clinically indicated, and subsequent CMR tissue characterization, can facilitate differentiation of ischemic and inflammatory myocardial injury. Early source control, appropriate antimicrobial therapy, hemodynamic support, and multidisciplinary coordination remain essential to achieving favorable outcomes.

### Conclusions

This case highlights the importance of considering acute myocarditis in patients with sepsis who develop peri-operative cardiovascular instability, elevated cardiac biomarkers, ischemic ECG changes, and acute LV dysfunction despite the absence of obstructive coronary artery disease. Acute myocarditis may mimic acute myocardial infarction and present as MINOCA, making diagnosis challenging. In our patient, coronary angiography excluded obstructive coronary disease, while CMR provided supportive evidence of myocardial inflammation and helped distinguish myocarditis from ischemic injury. The clinical context and temporal association with severe infection suggested a possible infectious trigger. Early source control, hemodynamic support, and multidisciplinary management were followed by improvement in cardiac function. This case suggests that acute myocarditis may be considered in septic patients with suspected ACS, with CMR playing a useful role in diagnostic evaluation and helping to guide management.

### Learning points

Acute myocarditis should be considered in septic patients with elevated cardiac biomarkers, ischemic ECG changes, and new LV dysfunction, particularly in the absence of obstructive coronary disease.

Acute myocarditis may present with a MINOCA-like clinical picture, making differentiation from acute myocardial infarction challenging.

Coronary angiography and CMR imaging can provide complementary diagnostic information, with angiography helping to exclude obstructive coronary disease and CMR helping to identify myocardial inflammation and support the diagnosis of myocarditis.

Early multidisciplinary management, including prompt infection source control, hemodynamic support, and appropriate heart failure therapy, may contribute to favorable clinical outcomes.

Timely recognition and appropriate supportive treatment may be associated with recovery of cardiac function and may help avoid unnecessary coronary intervention in selected patients.

## Data Availability

The authors declare that data supporting the findings of this study are available within the article.
